# Rapid, Selection-Free, High-Efficiency Genome Editing in Protozoan Parasites Using CRISPR-Cas9 Ribonucleoproteins

**DOI:** 10.1128/mBio.01788-17

**Published:** 2017-11-07

**Authors:** Lia Carolina Soares Medeiros, Lilith South, Duo Peng, Juan M. Bustamante, Wei Wang, Molly Bunkofske, Natasha Perumal, Fernando Sanchez-Valdez, Rick L. Tarleton

**Affiliations:** Center for Tropical and Emerging Global Diseases and Department of Cellular Biology, University of Georgia, Athens, Georgia, USA; Stanford University

**Keywords:** CRISPR, Cas9, *Leishmania*, SaCas9, *Trypanosoma*, *Trypanosoma brucei*, *Trypanosoma cruzi*, genome editing, ribonucleoproteins

## Abstract

Trypanosomatids (order Kinetoplastida), including the human pathogens *Trypanosoma cruzi* (agent of Chagas disease), *Trypanosoma brucei*, (African sleeping sickness), and *Leishmania* (leishmaniasis), affect millions of people and animals globally. *T. cruzi* is considered one of the least studied and most poorly understood tropical disease-causing parasites, in part because of the relative lack of facile genetic engineering tools. This situation has improved recently through the application of clustered regularly interspaced short palindromic repeats–CRISPR-associated protein 9 (CRISPR-Cas9) technology, but a number of limitations remain, including the toxicity of continuous Cas9 expression and the long drug marker selection times. In this study, we show that the delivery of ribonucleoprotein (RNP) complexes composed of recombinant Cas9 from *Staphylococcus aureus* (SaCas9), but not from the more routinely used *Streptococcus pyogenes* Cas9 (SpCas9), and *in vitro*-transcribed single guide RNAs (sgRNAs) results in rapid gene edits in *T. cruzi* and other kinetoplastids at frequencies approaching 100%. The highly efficient genome editing via SaCas9/sgRNA RNPs was obtained for both reporter and endogenous genes and observed in multiple parasite life cycle stages in various strains of *T. cruzi*, as well as in *T. brucei* and *Leishmania major*. RNP complex delivery was also used to successfully tag proteins at endogenous loci and to assess the biological functions of essential genes. Thus, the use of SaCas9 RNP complexes for gene editing in kinetoplastids provides a simple, rapid, and cloning- and selection-free method to assess gene function in these important human pathogens.

## IMPORTANCE

Protozoan parasites remain some of the highest-impact human and animal pathogens, with very limited treatment and prevention options. The development of improved therapeutics and vaccines depends on a better understanding of the unique biology of these organisms, and understanding their biology, in turn, requires the ability to track and manipulate the products of genes. In this work, we describe new methods that are available to essentially any laboratory and applicable to any parasite isolate for easily and rapidly editing the genomes of kinetoplastid parasites. We demonstrate that these methods provide the means to quickly assess function, including that of the products of essential genes and potential targets of drugs, and to tag gene products at their endogenous loci. This is all achieved without gene cloning or drug selection. We expect this advance to enable investigations, especially in *Trypanosoma cruzi* and *Leishmania* spp., that have eluded investigators for decades.

## INTRODUCTION

Clustered regularly interspaced short palindromic repeats–CRISPR-associated protein 9 (CRISPR-Cas9) technology is adapted from the bacterial immune system and has rapidly revolutionized the editing of many genomes. This system is based on two components: an RNA-guided endonuclease, classically the Cas9 protein, derived from the type II CRISPR-Cas system, and an RNA molecule that guides (gRNA) the nuclease to a complementary locus in the target DNA. Although the most frequently used Cas9 isoform was isolated from *Streptococcus pyogenes* (SpCas9), other Cas9 orthologues have been characterized. Among these, Cas9 isolated from *Staphylococcus aureus* (SaCas9) can edit genomes with efficiencies similar to those of SpCas9, while being nearly 40 kDa smaller (1).

The Cas9-gRNA ribonucleoprotein (RNP) complex can produce genome editing in a rapid, specific, and efficient manner (2). Most frequently, Cas9 or the Cas9-gRNA complexes are generated endogenously in transfected and marker-selected organisms. However, transgenic expression of Cas9 has demonstrated toxicity and instability in some cases (3–5), prompting the development and use of systems for regulating Cas9 expression (6–8). Alternatively, CRISPR-mediated editing can be achieved using Cas9 protein/gRNA complexes (termed RNP complexes below) generated *ex vivo* and delivered into cells by transfection. The rapid turnover of the RNP complexes within cells makes this approach transient, both overcoming the Cas9 toxicity issue (4, 9) and minimizing potential off-target edits in the genome (10).

Our group has focused on the development and application of techniques—including the CRISPR-Cas9 system—for functional analyses of genes in the protozoan parasite *Trypanosoma cruzi*, the cause of human Chagas disease (5, 11). Due to extensive genotypic and phenotypic variation within the species (12, 13), its complex genome, with a large number of very-high-copy-number multigene families (14–16), and the absence of tools for the facile manipulation of genes (e.g., unlike the related African trypanosome species, *T. cruzi* lacks functional RNA interference [RNAi] machinery), *T. cruzi* is considered one of the least studied and most poorly understood disease-causing pathogens.

To address some most of these challenges, we recently adapted the CRISPR-Cas9 system for *T. cruzi* (5), based on the delivery of *in vitro*-transcribed single guide RNA (sgRNA) into parasites selected for the stable expression of SpCas9. Using this approach, we demonstrated the rapid (within days) and highly eﬀicient knockout of single- and multicopy endogenous genes, including essential genes in both epimastigotes (life cycle stage present in the insect vector) and trypomastigotes (mammalian stage) of *T. cruzi*. Among the limitations of this approach were the low growth rate of SpCas9-expressing lines and the loss of Cas9 activity over time (5). An alternative *T. cruzi* CRISPR-based editing system developed by the Docampo laboratory required a long drug selection period (multiple weeks) and was not useful for the study of essential genes (17).

In this study, we show for the first time that RNP complexes of Cas9/gRNA can be delivered into epimastigotes and trypomastigotes of *T. cruzi*, with or without single-stranded repair oligonucleotides, to knock out or tag target genes with very high efficiency. The approach depends on the use of recombinant SaCas9 and is effective in all *T. cruzi* strains tested, as well as in the related trypanosomatids *T. brucei* and *Leishmania major*. These results open up a new avenue for genome editing in unicellular pathogenic parasites that are otherwise difficult to manipulate. This approach also offers a time-saving and potentially cloning-free methodology that could be used in virtually any trypanosome isolate and may also be applicable to other protozoans not evaluated herein.

## RESULTS

### SaCas9/sgRNA delivery and gene knockout in *T. cruzi* epimastigotes.

**FIG 1:**
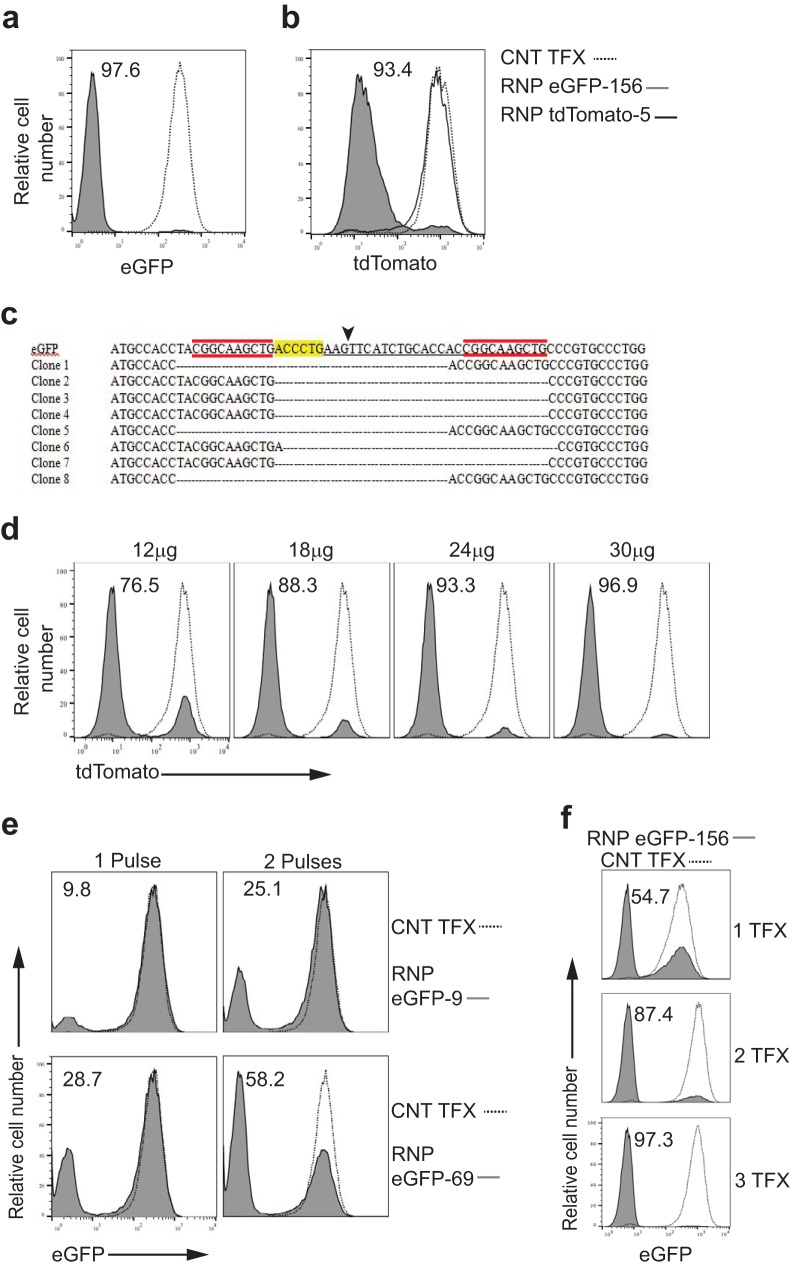
SaCas9/sgRNA delivery and gene knockout in *T. cruzi epimastigotes*. (a) Epimastigotes from *T. cruzi* expressing eGFP were electroporated with water (control transfection [CNT TFX]; dotted line) or RNP complexes containing SaCas9 and an sgRNA designated eGFP-156, targeting eGFP (RNP eGFP-156; gray filled) (see Table S1 in the supplemental material for description of all sgRNAs). (b) Epimastigotes from *T. cruzi* expressing tdTomato were electroporated with water (dashed line) or RNP complexes containing SaCas9 and sgRNA eGFP-156 (gray line) or an sgRNA designated tdTomato-5, targeting tdTomato (RNP tdTomato-5; grey filled). (c) DNA sequences of clonal lines from flow-sorted eGFP-negative parasites from the experiment whose results are shown in panel a. Arrowhead identifies the site of sgRNA eGFP-156-directed SaCas9 cleavage, highlighting identifies the PAM sequence, and red identifies microhomology regions. (d) Epimastigotes from *T. cruzi* expressing tdTomato were electroporated with increasing amounts of RNP complexes (SaCas9 and sgRNA tdTomato-5). The quantity of recombinant SaCas9 for each transfection is indicated (molar ratio of SaCas9/sgRNA is 1:1 in all cases). (e) Epimastigotes from *T. cruzi* expressing eGFP were electroporated with water (dashed line) or RNP complexes containing SaCas9 and sgRNA eGFP-9 or eGFP-69, targeting eGFP (gray filled). Parasites received 1 or 2 pulses of electroporation (the 2 pulses were applied consecutively). (f) Epimastigotes from *T. cruzi* expressing eGFP were electroporated with water (dashed line) or RNP complexes containing SaCas9 and an sgRNA targeting eGFP (eGFP-156; gray filled). Parasites received 1, 2, or 3 electroporations with a 2-day interval between each. Loss of eGFP or tdTomato fluorescence was determined 7 days after the first transfection. Each experiment was replicated 1 to 3 times with similar results.

To test the efficiency of gene editing of RNP complexes composed of SaCas9/sgRNA in *T. cruzi*, epimastigotes from *T. cruzi* strain CL expressing either enhanced green fluorescent protein (eGFP) or the tdTomato red fluorescent protein were transfected with RNPs targeting these transgenes, and fluorescent protein expression was assessed 7 days later. SaCas9/eGFP sgRNA complexes achieved >97% knockout efficiency, while spontaneous loss of GFP expression in control-transfected parasites was minimal (Fig. 1a). The editing specificity of the sgRNA complexed with SaCas9 was confirmed by electroporation of RNP complexes containing gRNAs targeting eGFP or tdTomato into tdTomato-expressing *T. cruzi*, achieving >93% knockout efficiency of tdTomato using the specific gRNA but no impact of SaCas9/eGFP sgRNA RNP complexes (Fig. 1b). DNA sequencing of the targeted eGFP site in clonal lines from flow-sorted eGFP knockout parasites showed a 33-base pair deletion at the sgRNA targeting site in all clones (Fig. 1c, arrowhead), a pattern consistent with microhomology-mediated end-joining (MMEJ) pathway repair of double-stranded breaks (DSBs) in *T. cruzi*, as previously described (5).

10.1128/mBio.01788-17.2 TABLE S1  sgRNA and repair templates used in the study. Download TABLE S1, PDF file, 0.2 MB. Copyright © 2017 Soares Medeiros et al. 2017 Soares Medeiros et al. This content is distributed under the terms of the Creative Commons Attribution 4.0 International license.

The knockout efficiency of RNP complexes in *T. cruzi* improved with increasing amounts of SaCas9/sgRNP complexes added (Fig. 1d). As previously reported for SpCas9 in *T. cruzi*, knockout efficiency varied depending on the choice of sgRNAs used (5), but gene knockouts with less-efficient RNP complexes could be substantially improved with the use of multiple transfection pulses (Fig. 1e), although usually at the cost of increased parasite death (not shown). Additionally, nearly complete gene knockout in a population could be achieved with lower-efficiency guides by serial transfection of RNP complexes at 2-day intervals (Fig. 1f).

### Cas9-RNP size impacts knockout efficiency.

**FIG 2:**
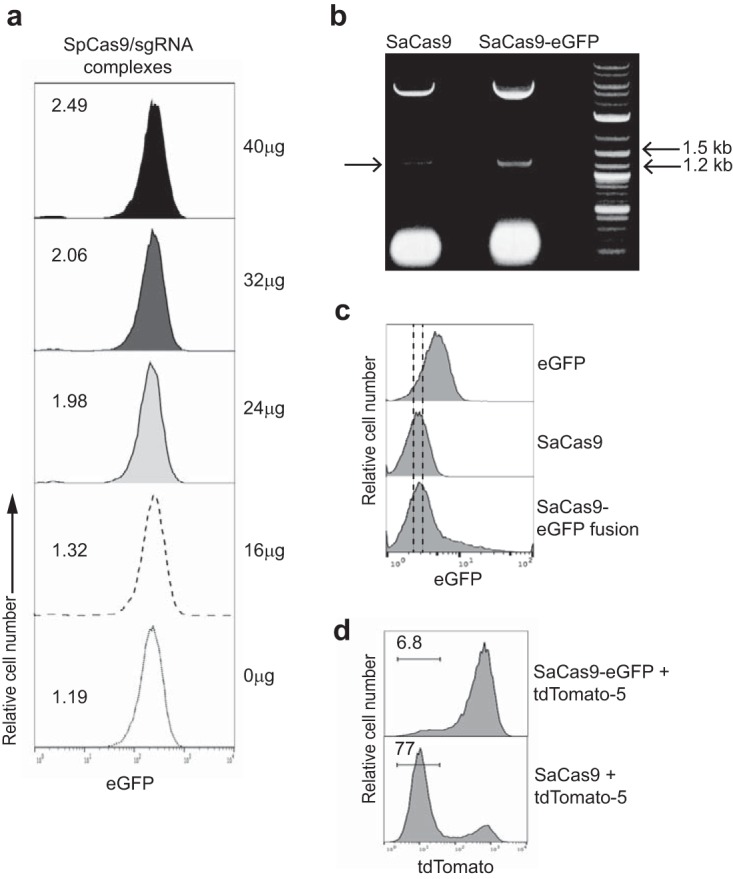
Cas9-RNP size impacts knockout efficiency. (a) Epimastigotes from *T. cruzi* expressing eGFP were electroporated with water (0 µg) or RNP complexes containing SpCas9 and sgRNA eGFP-153, targeting eGFP (sgRNA designed for association with SpCas9) in increasing amounts (as indicated, always keeping the molar ratio of SpCas9/sgRNA at 1:1). (b) Image of agarose gel showing cleavage product from *in vitro* nuclease activity assay performed with SaCas9 and SaCas9-eGFP fusion protein. Arrow on left indicates cleavage product. DNA markers are in the right lane. This experiment was performed twice, using the same batch of SaCas-eGFP fusion protein and different batches of SaCas9 protein. (c) Wild-type epimastigotes of *T. cruzi* were electroporated with eGFP, SaCas9, or SaCas9-eGFP fusion proteins. eGFP fluorescence was monitored immediately after electroporation by flow cytometry. A right shift in fluorescence of the entire parasite population in the eGFP-transfected group but not in those transfected with SaCas9-eGFP indicates the limited entry of SaCas9-eGFP into *T. cruzi* under these conditions. The experiment was performed 2 times with similar results. (d) Epimastigotes of *T. cruzi* expressing tdTomato were electroporated with RNP complexes containing SaCas9-eGFP or SaCas9 and sgRNA tdTomato-5 targeting tdTomato. Loss of tdTomato fluorescence was assessed 7 days posttransfection by flow cytometry. Loss of fluorescence was only observed in the parasites transfected with SaCas9 (approximately 124-kDa protein) and not in the parasites transfected with SaCas9-eGFP (153-kDa protein). The experiment was performed 2 times with similar results.

We had previously reported that electroporation of SpCas9 protein plus sgRNA into *T. cruzi* failed to achieve gene knockout (5), and we confirmed this result using increasing RNP concentrations similar to those shown to be effective for SaCas9-containing RNP complexes (Fig. 2a). In light of the results with SaCas9 reported here, we hypothesized that the mass difference between SaCas9 (1,053 amino acids [aa]; 124 kDa) and SpCas9 (1,368 aa; 163 kDa) might account for this difference in effectiveness. To test this hypothesis, we produced a fusion protein of SaCas9 linked to eGFP, creating a protein similar in size (1,310 aa; 153 kDa) to SpCas9 but with *in vitro* nuclease activity equivalent to that of nonfused SaCas9 (Fig. 2b). Applying the same electroporation conditions used to introduce SaCas9-containing RNP complexes into *T. cruzi*, recombinant eGFP could be detected in the cytoplasm of the majority of *T. cruzi* epimastigotes within 15 min after electroporation. In contrast, the eGFP fluorescence of the parasites electroporated with the SaCas9-eGFP fusion protein was only modestly different from that of controls receiving nonfluorescent SaCas9 (Fig. 2c). Moreover, while the SaCas9/sgRNA RNP provided highly efficient (>75% knockout of the tdTomato reporter), the SaCas9-eGFP-containing RNPs generated <7% tdTomato-less parasites (Fig. 2d). Thus, we conclude that there is a molecular mass limitation for efficient introduction of proteins and RNP complexes into *T. cruzi* that accounts for the differential ability to achieve genome editing with SaCas9 but not with the larger SpCas9 RNP complexes.

### SaCas9/sgRNA-mediated gene knockout in multiple strains and life cycle stages of *T. cruzi*.

**FIG 3:**
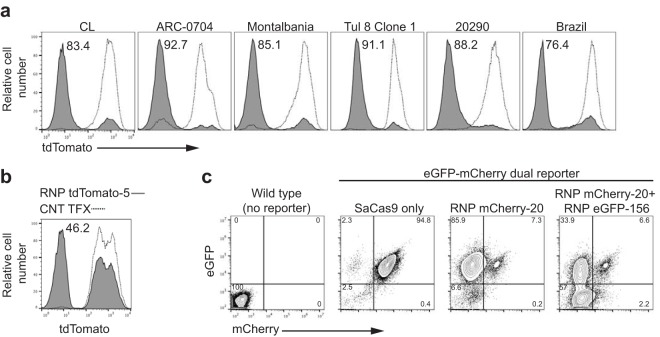
SaCas9/sgRNA-mediated gene knockout in multiple strains and life cycle stages of *T. cruzi*. (a) Epimastigotes of different *T. cruzi* strains, each expressing tdTomato, were electroporated with RNP complexes containing SaCas9 and sgRNA tdTomato-5, targeting tdTomato (gray filled). Negative controls were electroporated with water (dashed lines). Loss of tdTomato fluorescence was determined 7 days posttransfection by flow cytometry. (b) tdTomato-expressing trypomastigotes from *T. cruzi* strain CL were electroporated in the presence of either water (dashed line) or SaCas9/sgRNA tdTomato-5, targeting tdTomato (gray filled), and then incubated in flasks with a nearly confluent monolayer of noninfected Vero cells. Twenty-four hours after electroporation, the flasks containing Vero cells and trypomastigotes were washed to eliminate the parasites that had not adhered or invaded the host cells. Seven days posttransfection, trypomastigotes were harvested and loss of tdTomato fluorescence was determined by flow cytometry. (c) *T. cruzi* epimastigotes expressing mCherry and eGFP were electroporated with SaCas9 protein alone or with RNP complexes targeting only mCherry (>90% efficiency) or both mCherry and eGFP (~90% mCherry knockout and 55% double knockout). All experiments were replicated 2 times with similar results.

To validate the utility of RNPs to effect gene knockouts broadly in *T. cruzi*, we assessed the efficiency of disruption of tdTomato expression in parasite lines that spanned a range of genetic types (discrete typing units [DTU]), phenotypic characteristics (high and low virulence [strain ARC-0704 and strain Montalbania, respectively]), relative resistance to benznidazole (BZ) (strain ARC-0704), and length of time since isolation from naturally infected hosts (strains CL and Brazil are long-term laboratory strains, while strain 20290 was recently isolated from macaques in the United States). The tdTomato knockout frequencies were comparable among all six strains tested, varying between 76% for strain Brazil and 92% for strain ARC-0704 (Fig. 3a). Although less efficient than in epimastigotes, SaCas9/sgRNA RNP complexes also rapidly achieved gene knockouts in trypomastigotes of *T. cruzi* as well (Fig. 3b). Multiple genes can also be targeted in a single transfection, as shown by the ability to knock out both the mCherry red fluorescent protein and eGFP simultaneously by combining RNP complexes targeting each of these fluorescent-protein-encoding genes in dual reporter epimastigotes of *T. cruzi* (Fig. 3c).

### SaCas9/sgRNA-mediated homologous repair and essential gene knockouts.

**FIG 4:**
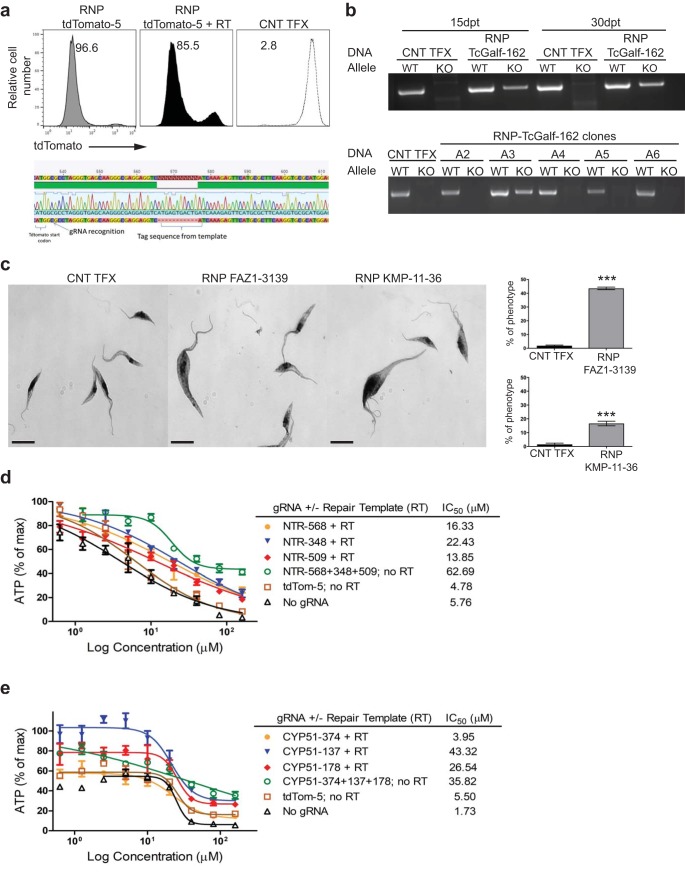
SaCas9/sgRNA-mediated homologous repair and knockout of essential genes. (a) *T. cruzi* epimastigotes expressing tdTomato were electroporated with water (CNT TFX; dashed line) or RNP complexes containing SaCas9/sgRNA tdTomato-5 (gray line) or this RNP complex plus a corresponding repair template which inserts a stretch of 3 in-frame stop codons into the tdTomato gene (grey filled). Sequencing of PCR products of the tdTomato gene from the tdTomato-negative population of the transfected parasites indicated successful insertion of 3 in-frame stop codons. The experiment was performed 3 times with similar results. (b) DNA isolated from uncloned (top) or cloned (bottom) *T. cruzi* epimastigotes (Brazil strain) that were transfected with water (CNT TFX) or with RNP complexes containing SaCas9 and an sgRNA designated TcGalf-162, targeting *T. cruzi* Galf (TcGalf) genes. WT, PCR result for wild-type allele; KO, PCR result for edited allele. Note that the results for day 15 and 30 uncloned epimastigote populations and clone A3 indicate the presence of both wild-type and edited alleles; other clones and control transfected parasites have wild-type alleles only. (c) Representative bright-field microscopy images of *T. cruzi* epimastigotes that were transfected with water (CNT TFX) or with RNP complexes containing SaCas9 and an sgRNA targeting FAZ1 (designated FAZ1-3139) or KMP-11 (designated KMP-11-36) genes. Bar graphs indicate the average percentage of parasites with a morphological defect/phenotype (e.g., greatly enlarged and multiflagellated parasites) for each group at 2 days (FAZ1) and 4 days (KMP-11) after transfection. Bar = 5 µm. Graphs are representative of 4 independent experiments, with data presented as mean values ± standard errors of the means (SEM). Faz1, 43.54 ± 0.4599, versus water control, 1.873 ± 0.2132; 95% confidence interval, −42.90 to −40.42. KMP-11, 16.60 ± 0.8253, versus water control, 1.466 ± 0.4684; 95% confidence interval, −17.46 to −12.81. ***, *P* < 0.0001, as determined by the unpaired, two-tailed Student *t* test. (d, e) Dose response curves of NTR (d) or CYP51 (e) knockdown epimastigotes under treatment with BZ (d) or POS (e). IC_50_ was determined as the drug concentration that was required to inhibit 50% of ATP production compared to that of parasites with no drug exposure (calculated with GraphPad Prism 5.00; GraphPad Software, Inc., San Diego, CA). Results are representative of 3 replicate experiments with similar outcomes.

We have previously shown that DNA cuts provided by endogenous Cas9 expression in *T. cruzi* are repaired by MMEJ or, preferentially, by homology-directed repair (HDR) when an appropriate repair template is provided (5). Codelivery of an RNP complex and a repair template containing stop codons in all three reading frames flanked by short homology regions resulted in gene knockout efficiencies similar to those achieved without the repair template, with confirmed insertion of the stop sequences (Fig. 4a). Including a sequence tag within the stop sequence-containing repair template allowed us to easily assess the knockout of endogenous genes by PCR. In the experiment whose results are shown in Fig. 4b, targeting two alleles encoding a conserved hypothetical protein (a possible triose-phosphate or UDP-galactofuranose transporter [Galf]) with an SaCas9/sgRNA complex, and a repair oligonucleotide including 3 in-frame stops and a short sequence from the commonly used M13 bacteriophage sequencing primer, revealed the generation of mutant alleles in the population 15 days after electroporation. Most clones from this population had only the wild-type (WT) allele for *Galf*. However, 3 of the 39 clones examined had both WT and mutant alleles, presumably one of each in this diploid organism. However, none were null mutants with both alleles edited (Fig. 4b). Similar results (see Fig. S1 in the supplemental material) were obtained in attempts to knock out the *T. cruzi* calreticulin (CRT) gene, a gene which has resisted previous attempts to generate null mutants using conventional gene knockout approaches (18). These results provide evidence of the ability to generate single-allele mutants using SaCas9-RNP when targeting genes that are apparently essential in *T. cruzi* but for which single-allele knockout is not lethal.

10.1128/mBio.01788-17.1 FIG S1  Generation of single-allele knockouts from an essential gene, the calreticulin (CRT) gene. *T. cruzi* Colombiana strain epimastigotes were electroporated with water (CNT TFX) or RNP complexes containing SaCas9 and an sgRNA targeting CRT genes (RNP CRT), plus a repair template containing stop codons in all three reading frames and the M13 sequence for use as a PCR anchor. (See Table S2 in the supplemental material for all primer sequences.) DNA was isolated from uncloned (top) or cloned (bottom) populations. Uncloned parasites (30 days posttransfection) showed evidence of both the wild-type and knockout alleles. However, clones from this population were either homozygous for the wild-type allele or heterozygous, with both wild-type and mutant alleles (clones B2 and B6). A total of 4 of the 20 clones examined presented both WT and mutant alleles, but none of the 20 had the mutant allele exclusively. Download FIG S1, EPS file, 1.5 MB. Copyright © 2017 Soares Medeiros et al. 2017 Soares Medeiros et al. This content is distributed under the terms of the Creative Commons Attribution 4.0 International license.

10.1128/mBio.01788-17.3 TABLE S2  Primers used in the study. Download TABLE S2, PDF file, 0.2 MB. Copyright © 2017 Soares Medeiros et al. 2017 Soares Medeiros et al. This content is distributed under the terms of the Creative Commons Attribution 4.0 International license.

The rapid and efficient knockout achieved with RNP complexes allowed us to also investigate the function of genes suspected or known to be essential and, thus, not amenable to previous knockout protocols that require an extended drug-selection period (17, 19, 20). Among such genes are those essential to the flagellar structure, as demonstrated using RNAi knockdown in *T. brucei* (21). Electroporation of RNP complexes targeting the *FAZ1* (22) and *KMP11* (23) genes results in a substantial population of epimastigotes with clear flagellar and cytokinesis defects within a few days after transfection (Fig. 4c).

A particularly useful application of gene knockdown is in the determination of the mode of action of antiparasitic drugs. Many parasite proteins involved in drug susceptibility are essential for viability; this is the case for the *T. cruzi* nitroreductase (NTR), required for the activation of the anti-*T. cruzi* compounds benznidazole (BZ) and nifurtimox (24, 25), and for lanosterol 14-alpha-demethylase (CYP51), the target of posaconazole (POS) (26). To determine the potential utility of RNP-mediated knockdown to identify putative drug targets, gRNAs and repair templates designed to truncate the products of the *NTR* and *CYP51* genes were transfected into epimastigotes with SaCas9, and 3 days later (to allow for loss of the targeted proteins in knockout parasites), these parasites were subjected to BZ or POS treatment. ATP production was used to determine parasite death/survival. With both genes, a single gRNA paired with a protein-truncating repair template or a combination of multiple gRNAs without repair templates (relying on MMEJ for deletion/truncation of the target gene) resulted in most cases in a 10-fold or greater increase in the 50% inhibitory concentration (IC_50_) (Fig. 4d and e). Thus, RNP-mediated knockdown of essential genes can be effectively used for the determination of gene/protein function and to confirm suspected targets of candidate drugs.

### SaCas9 RNP complexes for endogenous gene tagging.

**FIG 5:**
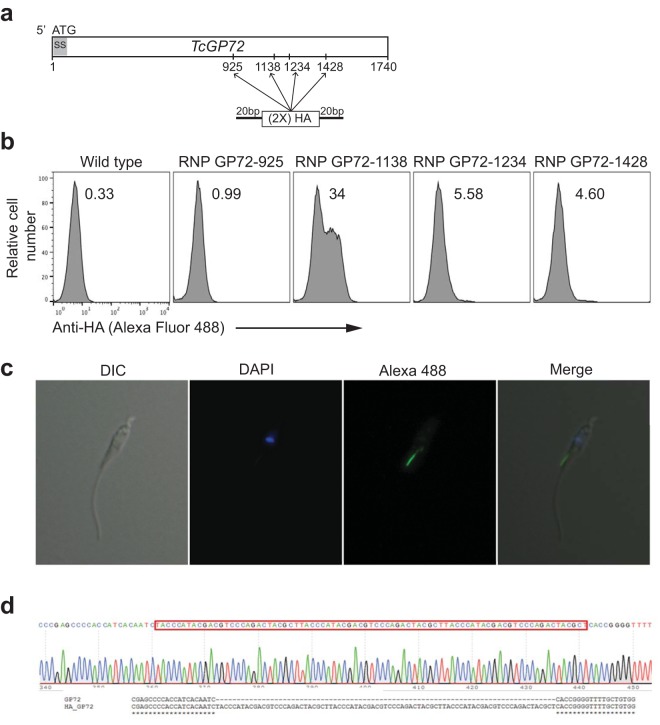
Endogenous gene tagging in *T. cruzi* using SaCas9 RNP complexes. (a) Four guide RNAs named with respect to the target nucleotide of the Cas9 cut site (sgRNA GP72-925, sgRNA GP72-1138, sgRNA GP72-1234, and sgRNA GP72-1428) were selected to cut the *T. cruzi* GP72 (TcGP72) gene at the indicated positions, avoiding the N- and C-terminal regions. ss, predicted signal sequence. For each guide, an in-frame DNA template was designed, containing 20-bp homology arms (each side) and two tandem sequences of the HA epitope. (b) Three days after electroporation, parasites were harvested by centrifugation and stained for detection of surface HA. Parasites receiving RNP complexes containing sgRNA GP72-1138 showed a shift in fluorescence for approximately 35% of the population, indicating incorporation of the HA tag. (c) The same parasites used in the experiment whose results are shown in panel b were adhered to poly l-lysine-coated coverslips, mounted in microscope slides, and observed via fluorescence microscopy. The HA tag fluorescence was observed in the flagellar attachment zone, as expected for GP72. DIC, differential inference contrast; DAPI, 4′,6-diamidino-2-phenylindole. (d) Sequencing of the GP72 gene in Alexa Fluor 488-positive parasites obtained from the experiment whose results are shown in panel b showed the correct insertion of the HA tag (in frame and in the expected position) in representative clones.

The ability to efficiently knock out gene function by the insertion of stop codons and sequence tags delivered with single-stranded repair templates suggested that SaCas9/sgRNA RNP-mediated cuts could also be used to insert expression tags into endogenous genes. To explore this possibility, we designed four gRNAs targeting sites within the *T. cruzi GP72* (*TcGP72*) gene and, for each gRNA, a corresponding DNA donor with *TcGP72* homology arms flanking 2 in-frame influenza hemagglutinin (HA) epitopes (Fig. 5a). Three days after the transfections, both control (water electroporated) and SaCas9/sgRNA-repair template-transfected parasites were stained with anti-HA antibodies and analyzed by flow cytometry. Approximately 35% of parasites receiving one of the four RNP-containing guides stained positive with anti-HA antibodies, indicating incorporation of the HA tag (Fig. 5b). Microscopic examination of these transfectants revealed localization of fluorescence on the parasite surface (in these nonpermeabilized parasites) and in the flagellar adhesion zone, as expected for *TcGP72* cellular localization (Fig. 5c) (27, 28). Sequencing of the gp72 target region in these parasites confirmed the in-frame insertion containing the HA epitopes (Fig. 5d) at a frequency (7 of 20 clones sequenced) similar to that detected by flow cytometry. Although we did not confirm insertion of the repair template in the cases of the other guide-template complexes that were not detected by flow cytometry (Fig. 5b), given the high efficiency of cut and repair observed in previous experiments, it is likely that the tag was inserted to some degree in all cases but was only accessible to detection when inserted in the position targeted by guide GP72-1138. Thus, rapid, high-efficiency endogenous tagging at non-N- and C-terminal sites and without drug selection is possible using RNP complexes.

### SaCas9/sgRNA delivery and gene knockout in the kinetoplastids *T. brucei* and *L. major*.

**FIG 6:**
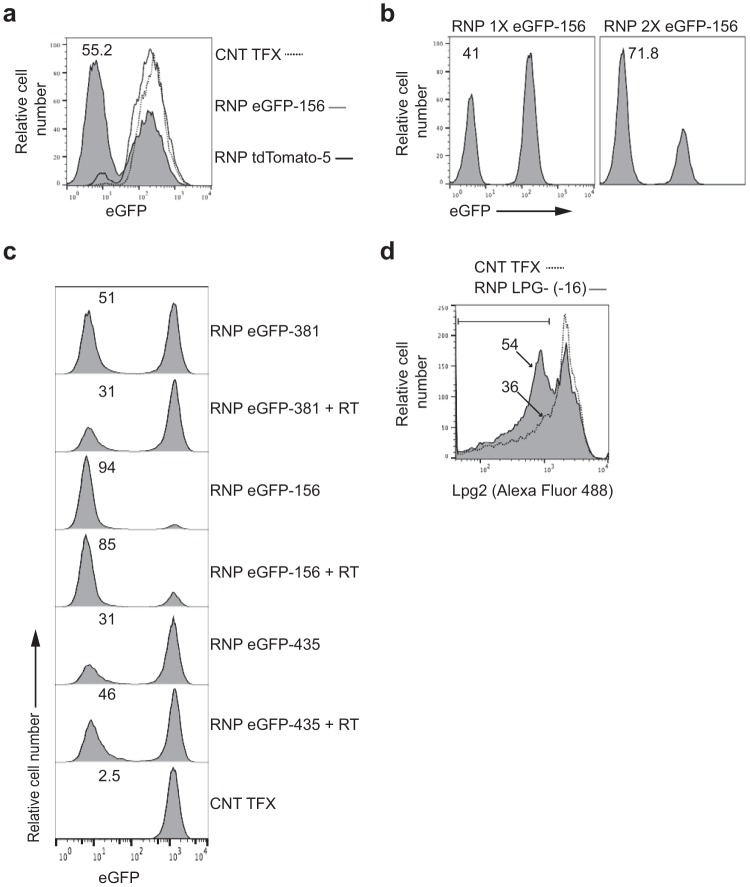
SaCas9/sgRNA delivery and gene knockout in the kinetoplastids *Trypanosoma brucei* and *Leishmania major*. (a) eGFP-expressing bloodstream forms of *T. brucei* were electroporated with one pulse in the presence of water (dashed line) or of RNP complexes containing SaCas9 and sgRNA eGFP-156, targeting eGFP (gray filled), or sgRNA tdTomato-5, targeting tdTomato (solid blackline). Loss of eGFP fluorescence was determined 5 days posttransfection by flow cytometry. (b) Five days after the first electroporation with SaCas9 and sgRNA eGFP-156, targeting eGFP, the mixed population of parasites was electroporated a second time with water (RNP 1× eGFP-156) or with the RNP complex targeting eGFP (RNP 2× eGFP-156). Loss of eGFP fluorescence was determined 5 days after the second electroporation by flow cytometry. The experiments whose results are shown in panels a and b were each performed once. (c) eGFP-positive *L. major* log-phase promastigotes were electroporated with RNP complexes containing SaCas9 and gRNAs targeting the eGFP gene at 3 positions (381, 156, and 435 bp downstream from the GFP start codon), with and without corresponding repair templates that insert a stretch of 3 in-frame stop codons at the gRNA target sites. Loss of eGFP fluorescence was determined 5 days posttransfection by flow cytometry. (d) *L. major* promastigotes were electroporated with RNP complexes containing SaCas9 and a gRNA targeting the *lpg2* gene, plus a corresponding repair template that inserts a stretch of 3 in-frame stop codons at the gRNA target site. Loss of parasite surface lipophosphoglycan was assessed at day 10 posttransfection by staining with WIC79.3 monoclonal antibody and Alexa Fluor 488 IgG secondary antibody. The experiments whose results are shown in panels c and d were replicated 2 and 4 times, respectively.

To test whether RNP complex delivery could be used to edit genes in other trypanosomatids, RNP complexes were delivered to eGFP-expressing bloodstream forms of *Trypanosoma brucei* and procyclic forms of *Leishmania major*. The RNP complex containing the same sgRNA used to knock out eGFP in *T. cruzi* epimastigotes (Fig. 1a) produced approximately 55% eGFP knockout in *T. brucei* bloodstream forms (Fig. 6a). As in the case of *T. cruzi*, additional RNP transfections, in this case 5 days after the initial transfection, substantially increased the knockout frequency (Fig. 6b). Knockout of an eGFP reporter in *L. major* was also achieved with gRNA-dependent efficiency in both the absence and presence of repair template (Fig. 6c). Lastly, disruption of the endogenous lipophosphoglycan (LPG) biosynthetic protein gene (*lpg2*; LmjF.34.3120) in *L. major* by SaCas9/sgRNA RNP complexes and a repair template resulted in the loss of surface phosphoglycans, as detected by staining with the LPG-specific WIC79.3 monoclonal antibody (Fig. 6d). Thus, SaCas9-containing RNP complexes have the ability to rapidly and efficiently edit genes in multiple kinetoplastid parasites.

## DISCUSSION

CRISPR-Cas9 has quickly developed into the method of choice for editing a wide variety of genomes, including those of unicellular eukaryotic pathogens which cause devastating human disease (5, 29–33). Kinetoplastids include human pathogens, such as *T. cruzi*, *T. brucei*, and *Leishmania* spp., that collectively infect tens of millions of humans worldwide. These diploid parasites have been challenging to genetically manipulate, requiring one or more rounds of time-consuming selection for the production of transgenics and null mutants. Only in *T. brucei* has RNAi been useful for determining gene essentiality and the mode of action of drugs and for genome-wide library screens (34–36). The machinery for RNAi is largely lacking in *T. cruzi* and *Leishmania* spp., and as a result, functional genetic studies have been much more limited in these pathogens.

CRISPR-Cas9 technology has been applied to *T. cruzi* (5, 17, 19, 20) and *Leishmania* (29, 37) and most recently to *T. brucei* (38). Even though very promising results have been reported in all these systems, significant issues remain. For example, all these approaches used gene cloning and drug selection processes to generate parasites expressing Cas9, an approach that can be time consuming and in some cases compromises parasite growth (5).

In this work, we demonstrate that Cas9-sgRNA RNP complexes induce rapid and efficient genome editing in kinetoplastids, with no apparent toxicity. The protocol functions in multiple species and in diverse isolates of the same species, as well as in different life cycle stages, and is effective for both reporter and endogenous (diploid) genes. Our approach allows gene mutations and functional knockout due to MMEJ-mediated deletions or when combined with repair templates, targeted truncations, and tagging of genes in their endogenous loci. These editing events can be achieved in a few days and do not require gene cloning or drug selection. Careful selection of gRNAs can provide nearly 100% editing efficiency, and the effectiveness of lower-performing gRNA can be enhanced by combining multiple gRNAs or by repeated electroporations. These factors combine to shave months off the process of generating gene knockouts or tagging in *T. cruzi* by avoiding the construction of plasmids and the need to select edited lines by drug resistance and/or by single-cell cloning and subsequent analysis (although clonal lines will, of course require a cell-cloning step). Furthermore, multiple genes can be edited simultaneously or serially (e.g., 3 or more/week) if desired.

The key to the success of the approach described herein was the choice to evaluate SaCas9 as the nuclease for the production of RNP editing complexes. Despite the widespread success of using SpCas9-sgRNA complexes to edit mammalian cell lines (9), our attempts to use SpCas9-RNP complexes for gene editing in *T. cruzi* failed (5). Previous studies suggested that molecular mass could significantly affect transfection efficiency in *T. cruzi*. For example, small proteins like eGFP (Fig. 2), as well as gRNAs and ~100-nucleotide oligonucleotide repair templates (5), can all be easily introduced into *T. cruzi* with very high efficiency, but larger proteins and plasmids enter with significantly lower efficiency. The description of the *Staphylococcus aureus* Cas9, which is smaller but has editing efficiency equivalent to that of the more commonly used *Streptococcus pyogenes* Cas9 (1), provided the opportunity to determine whether this size difference might allow SaCas9 RNP complexes to succeed where the SpCas9 RNP complexes had failed.

That protein (or protein-gRNA complex) size is the key for the high efficiency of SaCas9 RNPs is demonstrated by the failure of RNP complexes containing a SaCas9-eGFP fusion to effectively edit genes in *T. cruzi*. This sharp difference (eGFP and SaCas9 RNP complexes enter at up to 100% efficiency, while the 40- to 60-kDa-larger SaCas9-eGFP fusion protein and SpCas9 RNP complexes barely enter at all), rather than a more gradual decline in efficiency with increasing protein/RNP complex size, is interesting and may suggest a specific physical barrier to larger complexes. Fritz et al. (39) used the subpellicular microtubule network that comprises the trypanosome cytoskeleton to purify trypanosome RNP complexes by trapping them inside parasite cell ghosts formed by this microtubule network. It is possible that this same network acts to excludes proteins, RNP complexes, and plasmids beyond a certain size, perhaps accounting for the inefficiency of entry of these larger macromolecules into trypanosomes. It will be of interest in future studies to determine the specific size limitations for high-efficiency entry of proteins, DNA, and complexes into *T. cruzi* and whether temporary chemical destabilization of the microtubule network (40) might extend this range. The nearly 100% editing efficiency achieved using RNPs with selected sgRNAs may not be only about transfection efficiency, as it substantially exceeds that reported in mammalian cells using SpCas9-RNP complexes (9). We hypothesize that this high efficiency may also reflect the well-documented plasticity of the kinetoplastid genomes, wherein recombination is frequent and widespread (16) and is further facilitated by the targeted cuts introduced by Cas9.

Relative to SpCas9, SaCas9 recognizes a longer NNGRR(T) protospacer-associated motif (PAM) (1) that is expected to occur once in every 32 bp of random DNA, compared to the NGG PAM of SpCas9 (41, 42) that occurs once in every 8 bp. While potentially advantageous for specificity, the extended PAM sequence of SaCas9 translates into fewer potential editable sites per gene/genome (43). However, we have yet to find this to be an impediment for gene editing in *T. cruzi*. Additionally, an even smaller Cas9 derived from *Campylobacter jejuni* (CjCas9; 984 amino acid residues) has been identified and is able to induce targeted mutations at high frequencies in mouse muscle cells or retinal pigment epithelium cells (44) and could likely be used if the PAMs recognized by SaCas9 are found to be too infrequent.

The rapid and efficient knockdown of gene activity achieved with SaCas9 RNP complexes will be particularly useful for addressing the function of potential drug targets, as well as essential genes like those required for flagellar development and cytokinesis. This advance provides, for other kinetoplastids, a long-missing tool comparable in functionality to inducible RNAi in *T. brucei*.

We also demonstrate the versatility of RNP-mediated gene editing by epitope tagging the *TcGP72* gene at its endogenous locus. Tagging of genes at endogenous loci is preferred over the introduction of a tagged transgene, whose (often higher) expression level can substantially alter protein targeting and parasite viability. We chose GP72 as a target for tagging because its cellular localization is well characterized (27, 28). RNP complex delivery in the presence of a repair template containing a (2×)HA coding sequence resulted in the easily detectable tagging of GP72 at an internal site at a frequency of ~35% at 1 of the 4 selected target sites. No PCR amplifications, plasmid constructions, or drug selections were necessary, since the donor repair template can be synthesized as an ~100-bp oligonucleotide. Homology arms of ~20-bp flanking the (2×)HA coding sequence were sufficient for homology-directed repair. The simplicity of construction also allows one to easily test multiple potential tagging sites in a protein simultaneously.

These powerful gene-editing methods, initially developed for *T. cruzi*, were easily ported to two additional kinetoplastid species; we would expect this approach to also work with a range of other similar organisms. The wide within- and between-species applicability of these techniques, combined with the ease of production of RNPs using readily available reagents, opens up the possibility of gene manipulations to investigators working on virtually any kinetoplastid isolate.

## MATERIALS AND METHODS

### Parasites.

Epimastigotes of *T. cruzi* strain CL were cultured at 26°C in supplemented liver digest neutralized tryptose (LDNT) medium as described previously (11). Strains expressing eGFP or tdTomato were obtained as described previously (5) and maintained in the presence of 250 mg/ml G418 in the cultures. Trypomastigotes expressing tdTomato were maintained in Vero cells cultured in RPMI medium with 10% fetal bovine serum (FBS) at 37°C in an atmosphere of 5% CO_2_. *T. brucei* bloodstream forms expressing eGFP were a gift from Roberto Docampo (University of Georgia, Athens, GA) and were grown at 37°C in HMI-9 medium (45) supplemented with 10% FBS. *L. major* lines were the gift of Stephen Beverley (Washington University, St. Louis, MO) and were maintained in M199 medium.

### SaCas9 expression and purification.

The SaCas9 gene was obtained from the plasmid pSaCas9_GFP, a gift from Kiran Musunuru (Addgene plasmid number 64709), cloned with a nuclear localization signal into the pET-32 Ek/LIC vector (Novagen), and transformed into *E. coli* Rosetta 2(DE3) competent cells (Novagen). A bacterial preculture was grown overnight at 37°C with shaking and used to inoculate 100 ml of terrific broth medium (ratio of 1:100). The culture was grown at 37°C with shaking to an optical density at 600 nm (OD_600_) of 0.6 to 0.8. Protein expression was induced by the addition of isopropyl-β-d-thiogalactopyranoside (IPTG; 200 μM), and the culture was kept at 18°C with shaking overnight. Cells were harvested by centrifugation, and the pellet was resuspended in 40 ml lysis buffer containing 0.5 M NaCl, 20 mM Tris-HCl, pH 8, 5 mM imidazole, 5 mg lysozyme (Sigma), 3 tablets of cOmplete Mini EDTA-free protease inhibitor cocktail (Roche), DNase, RNase, and protease inhibitor cocktail (Sigma), and incubated for 1 h at 4°C. After lysis by sonication, the soluble fraction was obtained by centrifugation and purified by immobilized metal ion affinity chromatography (IMAC) using a Ni-nitrilotriacetic acid (NTA) chromatography cartridge (Thermo Scientific) in a fast protein liquid chromatography (FPLC) system (ÄKTAprime plus; GE Healthcare Life Sciences). All peak fractions were analyzed for the presence of SaCas9 using SDS-PAGE, and the purity was estimated to be ~80% based on band intensity as determined using ImageJ (version 1.51K, available at https://imagej.nih.gov/ij/index.html). After dialysis, the sample was concentrated to ~500 µl using a 50-kDa nominal molecular weight limit (NMWL) centrifugal concentrator (EMD Millipore). The protein concentration was determined using a bicinchoninic acid (BCA) protein assay kit (Thermo Scientific), and the yield was determined to be approximately 1.75 mg/100 ml of culture. Production can be scaled to at least 200-ml cultures, but attempts at fermentor-scale (5 to 10 liters) production resulted in poor yields.

### sgRNA preparation.

sgRNA target sequences and corresponding repair templates were identified using the Eukaryotic Pathogen CRISPR guide RNA/DNA Design Tool (http://grna.ctegd.uga.edu) (46) with the SaCas9 option (21-bp target sequence preceding an NNGRRT PAM site) and/or the SpCas9 option (20-bp target sequence preceding an NGG PAM site) (see Table S1 in the supplemental material). sgRNAs were prepared as described previously (5). Briefly, DNA templates for sgRNA *in vitro* transcription (IVT) were generated using PCR to amplify the sgRNA scaffold sequence specific for SaCas9 (1) or SpCas9, IVT was performed using the TranscriptAid T7 high-yield transcription kit (Thermo Scientific), and sgRNAs were purified by ethanol precipitation.

### SaCas9 and sgRNA assembly and electroporation.

SaCas9 and sgRNAs were prepared immediately before transfections. sgRNAs were annealed by heating to 90°C for 5 min and slowly cooling to room temperature. Equimolar amounts of SaCas9 and sgRNAs (1:1 ratio) were incubated at room temperature for 10 to 15 min. *T. cruzi* epimastigotes in early log phase, trypomastigotes harvested from Vero cell cultures, *T. brucei* bloodstream forms, and *Leishmania* promastigote forms in early log phase (1 × 10^6^ parasites) were resuspended in 100 µl room temperature human T cell Nucleofector solution (Amaxa AG). Twenty to 40 µg of DNA repair template was added when indicated. Electroporations were performed using an Amaxa Nucleofector device; program U-33 was used to electroporate *T. cruzi* and *Leishmania*, and program X-001 was used to electroporate *T. brucei* bloodstream forms. Electroporated *T. cruzi* epimastigotes were cultured in 25-cm^2^ cell culture ﬂasks (Corning, Inc.) with 5 ml LDNT medium. Electroporated *T. cruzi* trypomastigotes were cultured in flasks with a monolayer of noninfected Vero cells. Twenty-four hours after electroporation, the flasks containing Vero cells and trypomastigotes were washed to eliminate the parasites that had not invaded the host cells. *T. brucei* bloodstream forms were cultured in 25-cm^2^ cell culture ﬂasks with 5 ml HMI-9 medium. *Leishmania* promastigote forms were cultured in 25-cm^2^ cell culture ﬂasks with M199 medium.

### Flow cytometry.

The flow cytometry experiments were performed on live parasites, using a CyAn ﬂow cytometer (Beckman Coulter, Inc.), and data were collected by using Summit version 4.3 software (Beckman Coulter, Inc.) and analyzed with FlowJo (LLC). A minimum of 10,000 events were collected for parasites with a single fluorescence peak, and a minimum of 20,000 events were collected for fluorescent parasites with two or more fluorescence peaks. An empirical *T. cruzi* gate was applied to the collected events based on the forward and side scatterplot, and a singlet gate was also applied.

### SaCas9 and SaCas9-eGFP *in vitro* cleavage assay.

sgRNAs were annealed by heating to 90°C for 5 min and slowly cooling to room temperature. *In vitro* cleavage assays were performed by adding SaCas9 or SaCas9-eGFP (0.5 μg), sgRNA (5 μg), and cleavage buffer in a 20-μl final volume. After 15 min of incubation at room temperature, pTrex-eGFP plasmid DNA linearized using EagI-HF (high fidelity) was added to a final concentration of 10 ng/μl, and the mixture incubated at 37°C for 3 h. Reactions were terminated by the addition of proteinase K for 20 min at room temperature. The products of the reactions were loaded into a 1% agarose gel with ethidium bromide.

### Immunofluorescence.

Parasites were centrifuged, and the cell pellet was fixed in freshly prepared 4% (vol/vol) formaldehyde in PBS, followed by incubation in 3% (vol/vol) bovine serum albumin in PBS to reduce nonspecific protein binding. Parasites were incubated in a 1:600 dilution of the primary antibody against HA (Thermo Scientific) for 1 h at room temperature. After washing, a 1:1,000 dilution of Alexa Fluor 488 goat anti-rabbit IgG (Invitrogen) was used as a secondary antibody. Parasites were analyzed by flow cytometry or adhered to poly l-lysine-coated coverslips, mounted on microscope slides, and observed in a Leica fluorescence microscope.

### Transient knockdown of NTR and CYP51.

tdTomato-expressing *T. cruzi* strain Brazil epimastigotes in mid-log-phase growth were transfected with the RNP complexes indicated and 40 μg of repair template containing 3 in-frame stop codons (Table S1). The parasites were then cultured for 3 days to allow the expression of the targeted protein to decrease before exposure to BZ or POS for an additional 4 days. The viability of the parasites was determined by ATP production (ATPlite ATP luminescence detection assay system; PerkinElmer).

### Generation of endogenous gene knockouts.

*T. cruzi* epimastigotes of the Brazil strain in log-phase growth were transfected with RNP complexes containing sgRNAs designed to knock out presumptive GalF-encoding genes (TcCLB.511353.30 and TcCLB.511301.50), calreticulin (CRT) genes (TcCLB.509011.40 and TcCLB.510685.10), or the FAZ1 and KMP-11 flagellar protein-encoding genes, as well as 75 μg of repair template containing 3 in-frame stop codons (Table S1). For the GalF knockout, single parasites were flow sorted at 8 days posttransfection (d.p.t.) into individual wells of 96-well plates containing 50% LDNT medium and 50% parasite-conditioned medium. DNA from transfected parasites was isolated at 15 and 30 d.p.t. for the uncloned population and at 40 days postcloning to assess the genomic integration of the repair template using PCR. For genes encoding flagellar proteins, parasites were mounted on microscope slides and stained using a diff-quik Hema 3 stain kit (Fisher HealthCare Protocol Hema 3 fixative and solutions; Fisher Scientific). Slides were visualized using the Delta Vision microscope system I. Morphological phenotypes were quantified by counting >500 parasites per experimental group/replicate. For *lpg2* knockout in *L. major*, log-phase parasites were transfected with an RNP complex targeting the *lpg2* gene and the corresponding repair template to insert a stretch of 3 in-frame stop codons at the sgRNA target site. For detection of LPG expression, parasites were washed twice with and resuspended in a 1:2,000 dilution of WIC79.3 ascites fluid for 30 min with gentle shaking. Following two PBS washes, parasites were resuspended in a 1:3,000 dilution of Alexa Fluor 488 anti-mouse IgG secondary antibody (Molecular Probes; Invitrogen) for 30 min with gentle shaking. Single parasite cells were analyzed for fluorescence using flow cytometry. All staining steps were performed on ice or at 4°C.

### Statistical analyses.

Statistical analyses were performed using GraphPad Prism 5.00 (GraphPad Software, Inc., San Diego, CA). Analysis of the data was performed using the two-tailed, unpaired Student *t* test.

### Data availability.

The authors declare that all data supporting the findings of this study are available within the paper and its supplemental material.
