# Supplementary material for: Rapid, Selection-Free, High-Efficiency Genome Editing in Protozoan Parasites Using CRISPR-Cas9 Ribonucleoproteins
Source: mBio. 2017 Nov 7;8(6):e01788-17. doi: 10.1128/mBio.01788-17 (PMC5676044; doi:10.1128/mBio.01788-17)
Supplement: TABLE S1 [file mbo005173566st1.pdf]

gRNA name	target gene	GenomeDB ID	gRNA sequence	position in gene of target seq.	Cas9 used	repair template	use notes
eGFP-9	eGFP		GCAAGGGCGAGGAGCTGTTCAC/CGGGGT	9	SaCas9	n/a	knockout eGFP
eGFP-69	eGFP		GTACGTCGCCGTCCAGCTCGAC/CAGGAT	69	SaCas9	n/a	knockout eGFP
eGFP-156	eGFP		GCCGGTGGTGCAGATGAACTT/CAGGGT	156	SaCas9	GATGCCACCTACGGCAAGCTGACCCTGAAGTAGATAGATAGTTCATCTGCACCACCGGCAAGCTGCCCGTG	knockout eGFP
tdTomato-5	tdTomato		GCAAGGGCGAGGAGGTCATCA/AAGAGT	5	SaCas9	n/a	knockout tdTomato
eGFP-153	eGFP		GGTGGTGCAGATGAACTTCA/GGG	153	SpCas9	n/a	knockout eGFP using SpCas9
mCherry-20	mCherry		GAGGATAACATGGCCATCATCA/AGGAGT	20	SaCas9	n/a	knockout mCherry
Faz1-3139	FAZ1	TcCLB.509631.140	GAGCTTGAGCAGAAGGCAGCG/GAGAAT	3139	SaCas9	AGGCTGGCGGATGAGCTTGAGCAGAAGGCATAGATAGATAGGCGGAGAATGAGAGGCTGGCGGATGAGCTT	knockout FAZ1
KMP-11-36	KMP-11	TcCLB.510755.89	GAAGCTTGGCGGAGAATTCCTC/AAGAGT	36	SaCas9	TCTAAGTGAAAAATGGCCACCACTCTTGAGTAGATAGATAGGAATTCTCCGCCAAGCTTGACCGCCTGGAT	knockout KMP-11
TcGalf-162	Galf, putative	TcCLB.511353.30 and TcCLB.511301.50	GCCAAGGAACGTCATTACAAA/ATGAAT	162	SaCas9	GCCAGCACGACCCTCACTTTGATTCATTTTTAGATAGATAGTGTAAAACGACGGCCAGTGTAATGACGTTCCTTGGCCTTGTTTTTTGT	knockout GalF
NTR-568	nitroreductase	TcCLB.510611.60 and TcCLB.506791.70	GCAGATAGGCTGCGCCATAAA/GTGAGT	568	SaCas9	CTGGAGAGTGGGTACTACCACTCACTTTATTAGATAGATAGTGTAAAACGACGGCCAGTGGCGCAGCCTATCTGCGACATGCGTACTAC	knockout NTR
NTR-348	nitroreductase	TcCLB.510611.60 and TcCLB.506791.70	GTACCGCCCTGAACTTACAGCC/GTGGGT	348	SaCas9	GTACGGGCTCCTACCGCCCTGAACTTACAGTAGATAGATAGTGTAAAACGACGGCCAGTCCGTGGGTGGCTGTTGTCATACACGAGGAG	knockout NTR
NTR-509	nitroreductase	TcCLB.510611.60 and TcCLB.506791.70	GCGCTTGAGATGGGGCTGGAGA/GTGGGT	509	SaCas9	GCCCCGGCCGCGCTTGAGATGGGGCTGGAGTAGATAGATAGTGTAAAACGACGGCCAGTAGTGGGTACTACCACTCACTTTATGGCGCA	knockout NTR
CYP51-374	CYP51	TcCLB.506297.260 and TcCLB.510101.50	GAGCAGCTGAACTTTCTTGCGG/AGGAGT	374	SaCas9	AGGATGAGGGAGCAGCTGAACTTTCTTGCGTAGATAGATAGTGTAAAACGACGGCCAGTGAGGAGTTGACCATTGCGAAGTTTCAGAAC	knockout CYP51
CYP51-137	CYP51	TcCLB.506297.260 and TcCLB.510101.50	GTGCAGTTTGGCAAGAACCCGC/TTGAGT	137	SaCas9	GGACACATTGTGCAGTTTGGCAAGAACCCGTAGATAGATAGTGTAAAACGACGGCCAGTCTTGAGTTTATGCAGCGATGCAAACGTGAT	knockout CYP51
CYP51-178	CYP51	TcCLB.506297.260 and TcCLB.510101.50	GTGCATCGCTGCATAAACTCAA/GCGGGT	178	SaCas9	ATTGTGCAGTTTGGCAAGAACCCGCTTGAGTAGATAGATAGTGTAAAACGACGGCCAGTTTTATGCAGCGATGCAAACGTGATCTCAAG	knockout CYP51
GP72-925	GP72	TcCLB.509561.20	GCATGGGCCCATTGTTTCGCTT/GTGAAT	925	SaCas9	CACATGGGCCCATTGTTTCGTACCCATACGACGTCCCAGACTACGCTTACCCATACGACGTCCCAGACTACGCTCTTGTGAATATTAACAAACA	tag GP72 with HA tag
GP72-1138	GP72	TcCLB.509561.20	GAGCCCCACCATCACAATCCAC/CGGGGT	1138	SaCas9	CGAGCCCCACCATCACAATCTACCCATACGACGTCCCAGACTACGCTTACCCATACGACGTCCCAGACTACGCTCACCGGGGTTTTGCTGTGGC	tag GP72 with HA tag
GP72-1234	GP72	TcCLB.509561.20	GAAAGCAGTTCATGCATAAGCC/GCGGGT	1234	SaCas9	CAATCGAAGACCCGCGGCTTTACCCATACGACGTCCCAGACTACGCTTACCCATACGACGTCCCAGACTACGCTATGCATGAACTGCTTTCTTG	tag GP72 with HA tag
GP72-1428	GP72	TcCLB.509561.20	GCTCCTCTGAAGACATGCCCTC/TGGAGT	1428	SaCas9	CGGAATATTTTACTCCAGAGTACCCATACGACGTCCCAGACTACGCTTACCCATACGACGTCCCAGACTACGCTGGCATGTCTTCAGAGGAGGG	tag GP72 with HA tag
eGFP-435	eGFP		GTTGTACTCCAGCTTGTGCCC/CAGGAT	435	SaCas9	GACTTCAAGGAGGACGGCAACATCCTGGGGTAGATAGATAGCACAAGCTGGAGTACAACTACAACAGCCAC	knockout eGFP
eGFP-381	eGFP		CTTCAGCTCGATGCGGTTCAC/CAGGGT	381	SaCas9	GAGGTGAAGTTCGAGGGCGACACCCTGGTGTAGATAGATAGAACCGCATCGAGCTGAAGGGCATCGACTTC	knockout eGFP
LPG-(-16)	LPG2	LmjF.34.3120	AATTCTTTCGGTTGGGTTGCT/ACGGGT	-16	SaCas9	TTTCATCACAAAGCGAATCACCCGTAGCAATAGATAGATAGCCCAACCGAAAGAATTGCATCAGCAACTGT	knockout LPG2
TcCRT-181	CRT	TcCLB.509011.40 and TcCLB.510685.10	GAGCAAAGGTCTCCGTCTGACG/CGGGGT	181	SaCas9	GATGCGGAGAAGAGCAAAGGTCTCCGTCTGTAGATAGATAGTGTAAAACGACGGCCAGTACGGAGGATGCGCGGTTTTACGCCTTGTCT	knockout CRT

Column D:	Red, PAM sequences
Column G:	Blue, 3 frame stop codons
	Purple, M13 sequence
	Green, G manually added
	Black, homology arms
	Orange, 2xHA sequence
