# Supplementary material for: Rapid, Selection-Free, High-Efficiency Genome Editing in Protozoan Parasites Using CRISPR-Cas9 Ribonucleoproteins
Source: mBio. 2017 Nov 7;8(6):e01788-17. doi: 10.1128/mBio.01788-17 (PMC5676044; doi:10.1128/mBio.01788-17)
Supplement: TABLE S2 [file mbo005173566st2.pdf]

primer name	sequence	use
SaCas9_pET32_F:	GACGACGACAAGATGCCGAAGAAAAAGCGCAAGGTCGAAGCGTCCATGAAAAGG	cloning SaCas9 into pET
SaCas9_pET32_R:	GAGGAGAAGCCCGGTTAGCCCTTTTTGATAATCTGAGGGTGCTTTTTGCTCTTCAC	cloning SaCas9 into pET
SaCas9_sgRNA_F	GAGAATTGTAATACGACTCACTATAGGGAGAXXXXXXXXXXXXXXXXXXXXXGTTTTAGTACTCTGTAATTTTAGGTATGAGGTAGACGAAAATTGTA	sgRNA for SaCas9
SaCas9_sgRNA_R	AAAAAAATCTCGCCAACAAGTTGACGAGAT	sgRNA for SaCas9
SaCas9_scaff_F:	GTTTTAGTACTCTGTAATTTTAGGTATGAGGTAGACGAAAATTGTACTTATACCTAAAATTACAGAATCTACTAAAACAAGGCAAAATGCCGTGTTTA	sgRNA scaffold
SaCas9_scaff_R:	AAAAAAATCTCGCCAACAAGTTGACGAGATAAACACGGCATTTTGCCTTGTTTTAGTAGATTCTGTAATTTTAGGTATAAGTACAATTTTCGTCT	sgRNA scaffold
Forward primer (M13)	TGTAAAACGACGGCCAGT	test for insertion of M13 from repair template
Tc Galf reverse primer	GACCGGTGAAGTCTTCCCAA	test for Galf mutant and WT allele
TcWTGalf forward primer	TGATTCATTTTGTAATGACGTTCCT	test for Galf WT allele
TcCRT reverse primer	GTCATCCATCGTCTCCTCGT	test for CRT mutant and WT allele
TcCRT forward primer	AAGAGCAAAGGTCTCCGTCTGACG	test for CRT WT allele
